# Parity Affects the Gut Microbiome, Gut Homeostasis, and Glucose Metabolism in Aged Female Mice

**DOI:** 10.1093/geroni/igaf122.1796

**Published:** 2025-12-31

**Authors:** Megan Wong, Audrey Pierce, Kerry Wellenstein, Jennifer Lee

**Affiliations:** Tufts University School of Medicine, Medford, Massachusetts, United States; Tufts University School of Medicine, Medford, Massachusetts, United States; JM-USDA HNRCA at Tufts University, Boston, Massachusetts, United States; The JM-USDA Human Nutrition Research Center on Aging at Tufts University, Boston, Massachusetts, United States

## Abstract

Metabolic health declines with age. Parity, a history of prior reproduction, is implicated in regulating long-term metabolic health. Despite nearly 75% of women having a history of parity, virtually all preclinical studies use nulliparous mice, thus limiting the translation potential to improve women’s health. The gut microbiome and gut epithelium contribute to regulating host metabolism, but their roles in the context of parity remain unclear. Thus, this study aimed to determine parity effects on the gut microbiome, gut epithelium, and glucose metabolism in aged female mice. We metabolically phenotyped 16-21-month-old nulliparous and parous chow-fed C57BL/6 female mice. In the same mice, we sequenced the cecal metagenome, measured the cecal metabolome by mass spectrometry, and measured ileal expression of genes related to inflammation and cellular senescence. Compared with nulliparous mice, age-matched parous mice were more glucose tolerant despite having greater body weight and lean mass. Analyses of the cecal metagenome and metabolome identified several microbial species, including Lactobacillus reuteri, and specific metabolic pathways pointing to a unique signature of lipid metabolites, that were differentially regulated by parity. The expression of pro-inflammatory T-cell markers and hallmark cellular senescence genes were reduced by up to 10-fold in the ileum of parous mice versus nulliparous controls. Altogether, these data indicate an effect of parity on the gut microbiome and gut epithelium that likely regulates glucose metabolism in aged female mice. Identifying parity-dependent changes in the gastrointestinal tract may reveal novel gut-based targets with translational potential to better support women’s health outcomes across the lifespan.

